# Physiologic intestinal ^18^F-FDG uptake is associated with alteration of gut microbiota and proinflammatory cytokine levels in breast cancer

**DOI:** 10.1038/s41598-019-54680-3

**Published:** 2019-12-04

**Authors:** Hai-Jeon Yoon, Han-Na Kim, Ji-In Bang, Woosung Lim, Byung In Moon, Nam Sun Paik, Bom Sahn Kim, Hyung-Lae Kim

**Affiliations:** 1 Department of Nuclear Medicine, Ewha Womans University School of Medicine, Seoul, Republic of Korea; 2 Medical Research Institute, Kangbuk Samsung Hospital, Sungkyunkwan University, School of Medicine, Seoul, Republic of Korea; 3 Department of Clinical Research Design and Evaluations, SAIHST, Sungkyunkwan University, Seoul, Republic of Korea; 4 Department of Surgery, School of Medicine, Mokdong Hospital, Ewha Womans University, Seoul, Republic of Korea; 5 Department of Biochemistry, Ewha Womans University, School of Medicine, Seoul, Republic of Korea

**Keywords:** Bacterial genes, Breast cancer

## Abstract

The clinical significance of physiologic Fluorine-18-fluorodeoxyglucose (^18^F-FDG) intestinal uptake (IU) based on the predicted link with gut microbiota dysbiosis and inflammatory cytokine production was investigated in a cohort of breast cancer patients. A total of 114 patients were visually classified into the lower or higher IU group. The maximum and mean standardized uptake values of total bowel (TB SUV_max_ and TB SUV_mean_) were measured. The gut microbial abundance of the *Citrobacter* genus of the Enterobacteriaceae family showed a significant positive correlation with TB SUV_max_ and TB SUV_mean_ (*q* = 0.021 and *q* = 0.010). The unclassified Ruminococcaceae showed a significant negative correlation with TB SUV_max_ (*q* = 0.010). The level of tumor necrosis factor alpha (TNF-α) was significantly increased in the high IU group (*p* = 0.017). The TNF-α levels showed a significant positive correlation with TB SUV_max_ (*rho* = 0.220 and *p* = 0.018) and TB SUV_mean_ (*rho* = 0.250 and *p* = 0.007). Therefore, our findings suggest that the physiologic intestinal uptake may reflect subclinical inflammation and differences in the composition of the gut microbiome in breast cancer patients.

## Introduction

Fluorine-18-fluorodeoxyglucose (^18^F-FDG) positron emission tomography (PET) is important for the accurate staging and management of breast cancer. Though this modality has been widely used for the evaluation of cancer, applications for PET have expanded to non-cancer pathophysiology.

The intestine, which is involved in diverse metabolic pathways, presents a variable range of ^18^F-FDG uptakes (also referred to as the “physiologic uptake”) regardless of pathologic lesions. The mechanism and clinical significance of physiologic intestinal uptake are not fully understood. Recently, several studies have suggested that the gut microbiota may play a role in the physiologic intestinal uptake^1–4^.

The gut microbiota is a community consisting of trillions of microorganisms residing along the intestinal tract. The low or high abundance of the specific microorganisms within the community is known to induced aberrant immune responses via the stimulation of monocyte-derived proinflammatory cytokines^5,6^. Moreover, it has been hypothesized that a crosstalk between microbiota and estrogen synthesis occurs, indicating that microbial dysbiosis may be associated with developing hormone-related breast cancer^7,8^.

Recently, Kang *et al*. reported that the physiologic intestinal uptake of ^18^F-FDG was associated with different abundances of specific intestinal bacteria in healthy subjects^2^. They postulated that the mucosal inflammation, impaired barrier function and gut permeability caused by the dysbiosis contributes to physiologic intestinal uptake. However, their work did not clearly demonstrate a link between mucosal inflammation and physiologic intestinal uptake.

Our previous study found that increases in physiologic intestinal uptake are correlated to the serum lipid profile and obesity in breast cancer patients^4^. We also postulated that alterations in the composition of the gut microbiota may affect lipid metabolism and physiologic intestinal uptake. However, the details of the relationship between the gut microbiota and the physiologic intestinal uptake could not be identified. Therefore, in this study, we investigated the relationship between the composition of gut microbiota with the range of physiologic intestinal uptakes by using high-throughput sequencing of the 16S rRNA gene in breast cancer subjects. Furthermore, the relationship of tumor necrosis factor alpha (TNF-α) and interleukin-1 (IL-1) with the range of physiologic intestinal uptakes were also investigated. With this study, we aimed to elucidate the clinical significance of physiologic intestinal uptake based on the predicted link with gut microbial dysbiosis and inflammatory cytokine production in a breast cancer cohort.

## Materials and Methods

### Subjects

Participants were recruited from the patients who visited our institutional PET/CT Center for their initial breast cancer staging work up. Participants who met the following inclusion criteria were enrolled in this study: (1) Patients should not have a medication history that includes lipid-lowering drugs or probiotics taken within four weeks of enrollment, as these drugs can influence the gut microbiota; (2) Patients should not have a medication history of Metformin, which can influence intestinal FDG uptake; (3) Patients should not have undergone neoadjuvant chemotherapy before PET/CT; (4) Patients who do not have concurrent inflammatory bowel disease, infectious colitis, etc., or have taken any antibiotics within six weeks of enrollment. Stool and blood samples were collected from 121 female participants between the ages of 32 and 78 who underwent a PET/CT scan between April 2016 and February 2017. Among the participants, seven patients whose samples had undetectable cytokine levels were excluded.

Demographics, laboratory data, and other clinical data were extracted from the medical chart, and questionnaires were taken at the time of the initial visit. This study protocol was approved by the Institutional Review Board of Ewha University Medical Center (2016-01-017-002). The informed consents were obtained from all participants of the study. We followed all applicable institutional and governmental regulations concerning the ethical use of human volunteers during the study.

### DNA extraction from fecal samples

Fecal samples were frozen immediately after defecation at −20 °C and were placed at −70 °C within 24 hours. DNA extraction from the fecal samples was performed within one month by using the MOBio PowerSoil® DNA Isolation Kit (MO BIO Laboratories, Carlsbad, CA) following the manufacturer’s instructions.

### PCR Amplification and sequencing of bacterial 16S rRNA gene

Variable V3 and V4 regions of the 16S rRNA gene were amplified with the universal primers 341F (5′ TCG TCG GCA GCG TCA GAT GTG TAT AAG AGA CAG CCT ACG GGN GGC WGC AG 3′) and 805R (5′ GTC TCG TGG GCT CGG AGA TGT GTA TAA GAG ACA GGA CTA CHV GGG TAT CTA ATC C 3′), with each primer modified to contain a unique 8 nt barcode index by combination of Nextera XT DNA Library Preparation kit (Illumina, San Diego, CA). PCR reactions contained 5 ng/μL DNA template, 2 × KAPA HiFi HotStart Ready Mix (KAPA Biosystems, Wilmington, MA) and 2 pmol of each primer. Reaction conditions consisted of an initial incubation at 95 °C for 3 min, followed by 25 cycles of 95 °C for 30 s, 55 °C for 30 s, and 72 °C for 30 s. Samples were subjects to a final extension incubation at 72 °C for 5 min. After PCR clean-up and index PCR, sequencing was performed on the Illumina MiSeq platform following the manufacturer’s specifications^9,10^.

### 16S rRNA gene compositional analysis

The DADA2 pipeline within the QIIME2 package (https://qiime2.org) was used to filter out low quality and chimera errors and generate unique sequence variants^11,12^. The amplicon sequence variants (ASVs) were produced by denoising with DADA2 and regarded as 100% operational taxonomic units (OTUs). QIIME2 constructed the Feature Table, which is the equivalent of the biome table and the representative sequence file. The sequences were mapped at 99% sequence identity to an optimized version of the GreenGenes database (version 13.8) containing the V3-V4 region to determine taxonomies.

### Serum cytokine analysis

Serum concentration of TNF-α and IL-1 was measured by an enzyme-linked immunosorbent assay (ELISA). Briefly, 96-well microtiter plates were coated with the capture antibody (human IL-1β or TNF-α capture; R&D Systems) and were incubated at 4 °C overnight, then treated with blocking buffer at room temperature. Diluted test samples and standard recombinant proteins (human IL-1β or TNF-α; R&D Systems) were added to each well, followed by incubation with the biotinylated detection antibody (human IL-1β or TNF-α detection; R&D Systems). After the incubation, Streptavidin-Horseradish Peroxidase (Thermo Scientific) was added to the plates, followed by the TMB Substrate Solution (Thermo Scientific) for 30 minutes. The reaction was stopped by addition of the stopping solution and the absorbance was measured at 450 nm in a VersaMax microplate reader (Molecular Devices, LLC, USA).

### ^18^F-FDG PET/CT and image analysis

For the ^18^F–FDG PET/CT analysis, patients were instructed to fast for at least six hours before intravenous FDG administration (3.7 MBq per kg) and then to rest for one hour before the scan. Fasting blood glucose levels were measured before FDG administration and confirmed to be <140 mg/dL. A CT scan without contrast agent was obtained first, and then a PET scan was obtained from the skull base to the mid-thigh, using a Siemens Biograph mCT with 128 CT slices (Siemens Medical Solutions, Erlangen, Germany). Low-dose CT was acquired under a CARE Dose system (Siemens Medical Solutions, Erlangen, Germany) and CT-based attenuation correction was performed. The spatial resolution at the center of the PET was 2.0 mm full width at half maximum (FWHM) in the trans-axial direction and 2.0 mm FWHM in the axial direction. PET scans were acquired for 2 min per bed position (5–7 positions) with 3D emission mode. PET images were reconstructed to 200 × 200 matrices and 3.4 mm × 3.4 mm pixel size with 3.0 mm slice thickness using a 3D-OSEM iterative algorithm (2 iterations and 21 subsets) with time of flight (TOF) and point spread function (PSF).

The physiologic intestinal FDG uptake (IU) was assessed visually and quantitatively by two independent nuclear medicine specialists. The analysis was carried out in consensus, and the interpreters were strictly blinded to clinical and laboratory data. For visual assessment, the range of IU was dichotomized as low and high. Briefly, patients with lower FDG uptake of the whole intestine compared to that of the liver were classified as the lower IU group, while patients with FDG uptake of at least one bowel segment greater than that of the liver were classified as the higher IU group. For quantitative assessment, the maximum and mean standardized uptake values (SUV_max_ and SUV_mean_) in different bowel segments were measured by placing a three-dimensional volume of interest (VOI)^2,4^. The SUV_mean_ was measured with a margin threshold of 40% SUV_max_. For the precise measurement of IU, the placement of VOI was carefully determined and adjusted to avoid possible spillover uptake from neighboring tissue and intraluminal stool by simultaneous review of PET and CT slice. The small bowel IU was measured at the third duodenum, jejunum, and distal ileum loop. The large bowel IU was measured at the cecum, hepatic flexure, splenic flexure, and descending colon-sigmoid junction. Then, SUVs of different bowel segments were averaged for a measure of the total bowel IU (TB SUV_max_ and TB SUV_mean_).

### Statistical analysis

Basic statistical analyses were performed using the MedCalc software. All data were presented as mean ± standard deviations. Exploratory and differential microbial composition analyses were conducted in QIIME2 (version 2017.12). Microbial richness, which measures the number of distinct ASVs in each sample, was measured based on the actual number of different taxa observed in a sample (“Observed ASVs”). The alpha diversity was also measured with two non-phylogenetic methods, the Shannon index, which is measured by accounting for both evenness and richness^13^, and the Pielou’s evenness, which quantifies how equal the community is numerically^14^. Additionally, Faith’s PD index was measured to find phylogenetic difference between ASVs^15^. The Kruskal-Wallis test was used to examine the differences between groups in the alpha diversity. For the analysis of beta diversity, the dissimilarity between groups was calculated as unweighted and weighted UniFrac^16^ values for phylogenetic differences and compared between the IU groups using pairwise Permutational Multivariate Analysis of Variance (PERMANOVA) with 999 permutations^17^. A resulting *p*-value of less than 0.05 was considered statistically significant.

The abundance of taxa was compared between IU groups and correlated with TB SUV_max_, as well as TB SUV_mean_ by the generalized linear models implemented in Multivariate Association with Linear Models (MaAsLin) packages (https://huttenhower.sph.harvard.edu/maaslin) of RStudio (version 0.98.983), considering the effects of other variables (confounders) in the study population (i.e., age and BMI)^18^. All analyses in MaAslin were performed using the default options. The resulting *p*-values were corrected for multiple comparisons on each phylogenetic level using Benjamini-Hochberg correction (FDR). A *q*-value less than 0.05 was considered statistically significant.

## Results

### Patient characteristics

The baseline characteristics of 114 patients are summarized in Table 1. Based on the visual grade of intestinal FDG uptake (IU), 75 participants (65.8%) were classified in the lower IU group, while 39 participants (34.2%) were classified in the higher IU group. The binary logistic regression analysis showed that none of the demographic and laboratory features we measured had a significant relationship with visually graded IUs (Table 2). Based on the quantitative assessment of IU, the overall TB SUV_max_ was 1.99 ± 0.57 (range 0.59–5.51) and TB SUV_mean_ was 1.65 ± 0.42 (range 0.48–3.94). According to the linear regression analysis, none of demographic and laboratory features measured had a significant correlation to the TB SUV_max_ (Table 3), or the TB SUV_mean_ (Table 4).

**Table 1:** General Characteristics.

	Mean ± SD (range)
Age (years)	50.26 ± 9.09 (32.00–78.00)
Body mass index (kg/m^2^)	23.61 ± 3.19 (14.40–31.60)
DM, n (%)	3 (2.63)
Hypertension, n (%)	14 (12.28)
Smoking status, n (%)	11 (9.65)
White blood cell (103/mm^3^)	6.38 ± 1.90 (2.95–14.22)
Neutrophil (%)	59.61 ± 9.98 (36.30–83.20)
Lymphocyte (%)	30.74 ± 8.59 (4.70–51.90)
Monocyte (%)	5.50 ± 3.80 (0.80–34.80)
Platelet (109/L)	261.18 ± 55.78 (148.00–447.00)
Hematocrit (%)	39.55 ± 2.65 (31.10–44.70)
Fasting glucose level (mg/dL)	99.39 ± 13.21 (75.00–173.00)
Triglyceride (mg/dL)	125.20 ± 71.24 (32.00–418.00)
Cholesterol (mg/dL)	186.48 ± 39.99 (34.00–313.00)

**Table 2:** Binary logistic regression analysis according to IU group.

Variables	p value	Odds ratio	95% CI
Age (years)	0.651	1.010	0.968–1.054
Body mass index (kg/m^2^)	0.946	1.004	0.889–1.134
White blood cell (103/mm^3^)	0.567	1.061	0.867–1.298
Neutrophil (%)	0.676	1.008	0.970–1.048
Lymphocyte (%)	0.923	0.998	0.954–1.044
Monocyte (%)	0.150	0.837	0.656–1.067
Platelet (109/L)	0.102	1.006	0.999–1.013
Hematocrit (%)	0.045	0.858	0.738–0.997
Fasting glucose level (mg/dL)	0.526	0.990	0.960–1.021
Triglyceride (mg/dL)	0.845	1.001	0.995–1.006
Cholesterol (mg/dL)	0.112	0.992	0.981–1.002

**Table 3:** Linear regression analysis according to TB SUV_max_.

Variables	p value	HR	95% CI
Age (years)	0.143	0.009	−0.003–0.020
Body mass index (kg/m^2^)	0.073	0.030	−0.003–0.063
White blood cell (103/mm^3^)	0.725	0.010	−0.046–0.066
Neutrophil (%)	0.923	−0.001	−0.011–0.010
Lymphocyte (%)	0.970	0.000	−0.012–0.013
Monocyte (%)	0.547	−0.008	−0.036–0.019
Platelet (109/L)	0.203	0.001	−0.001–0.003
Hematocrit (%)	0.413	−0.017	0.057–0.023
Fasting glucose level (mg/dL)	0.111	0.006	−0.001–0.014
Triglyceride (mg/dL)	0.420	0.001	−0.001–0.002
Cholesterol (mg/dL)	0.184	−0.002	−0.005–0.001

**Table 4:** Linear regression analysis according to TB SUV_mean_.

Variables	p value	HR	95% CI
Age (years)	0.336	0.004	−0.004–0.013
Body mass index (kg/m^2^)	0.246	0.015	−0.010–0.039
White blood cell (103/mm^3^)	0.662	0.009	−0.033–0.051
Neutrophil (%)	0.999	0.000	−0.008–0.008
Lymphocyte (%)	0.889	−0.001	−0.010–0.009
Monocyte (%)	0.598	−0.006	−0.026–0.015
Platelet (109/L)	0.259	0.001	−0.001–0.002
Hematocrit (%)	0.402	−0.013	−0.043–0.017
Fasting glucose level (mg/dL)	0.166	0.004	−0.002–0.010
Triglyceride (mg/dL)	0.622	0.000	−0.001–0.001
Cholesterol (mg/dL)	0.122	−0.002	−0.004–0.000

### Comparison of alpha and beta diversity within and between the intestinal FDG uptake groups

**Figure 1:**
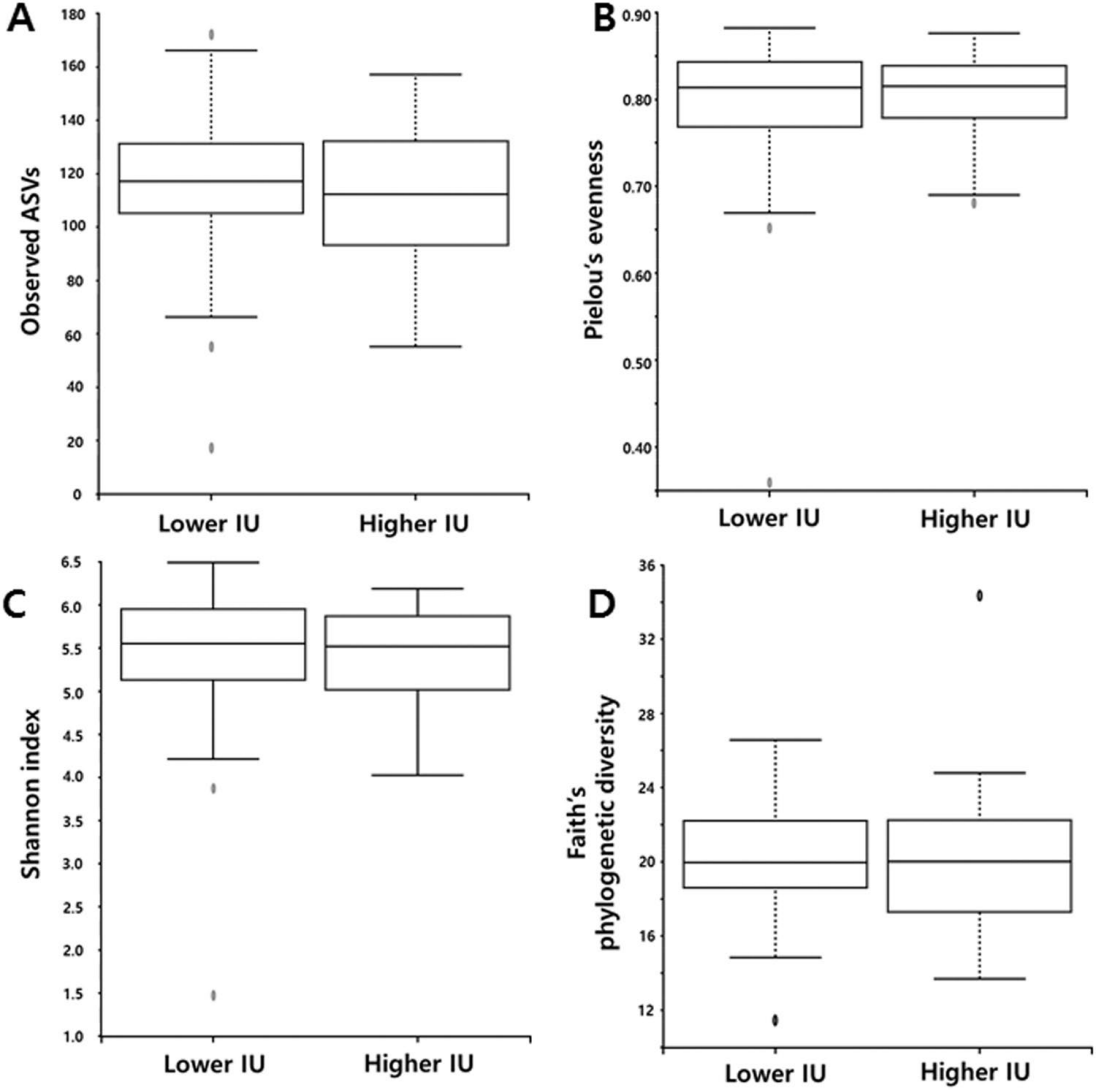
Comparison of alpha diversity between lower and higher IU group by the Observed ASV (**A**), Evenness (**B**), Shannon index (**C**), and Faith’s phylogenetic diversity (**D**) method.

**Figure 2:**
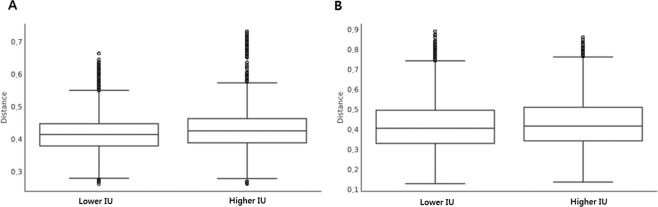
Distance of beta diversity from the lower to higher IU group by unweighted UniFrac-based (**A**) and weighted UniFrac-based (**B**) analysis.

The 16S RNA gene sequencing produced 3,809,809 sequences across 114 samples (mean of 31,266 sequences per sample). The alpha diversity of gut microbial taxa between the lower IU and higher IU groups did not show statistically significant differences in observed ASVs (116.13 ± 27.03 vs. 1092.35 ± 26.64, *p = *0.235), evenness (0.80 ± 0.07 vs. 0.80 ± 0.05, *p* = 0.983), Shannon’s index (5.46 ± 0.71 vs. 5.41 ± 0.57, *p* = 0.498), and Faith’s PD (20.13 ± 2.56 vs. 20.04 ± 3.71, *p* = 0.682) (Fig. 1). Unweighted UniFrac-based beta diversity, which is a phylogenetic and qualitative index, demonstrated that there were no significant differences in distance between the lower IU and higher IU group (*p* = 0.102; Fig. 2A), while weighted UniFrac-based beta diversity, which is a phylogenetic and quantitative index, showed marginal significance (*p* = 0.062; Fig. 2B).

### Association between gut microbial taxa composition and intestinal FDG uptake

In the analysis of gut microbial abundance of patients in each of the visually graded IU groups, the genus *Enterobacter*, which belongs to the family Enterobacteriaceae, showed a trend of higher relative abundance in high IU group compared with low IU group (*CE* = 1.08; *p* < 0.001; *q* = 0.094; Supplement Fig. S1A). Conversely, the unclassified Ruminococcaceae trended towards being in lower relative abundance in the high IU group compared to the low group (*CE* = −0.386; *p* < 0.001; *q* = 0.083; Supplement Fig. S1B).

**Figure 3:**
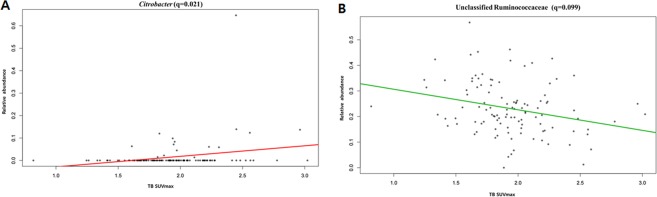
Correlations of the TB SUV_max_ with *Citrobacter* (**A**) and the unclassified Ruminococcaceae (**B**).

Analyzing the correlation between TB SUV_max_ and gut microbial abundance after adjusting for age and BMI, the *Citrobacter* genus showed a significant positive correlation with TB SUV_max_ (*CE* = 2.26; *p* < 0.001; *q* = 0.021; Fig. 3A), while the unclassified Ruminococcaceae showed a negative association with the TB SUV_max_ (*CE* = −0.536; *p* < 0.001; *q* = 0.099; Fig. 3B).

**Figure 4:**
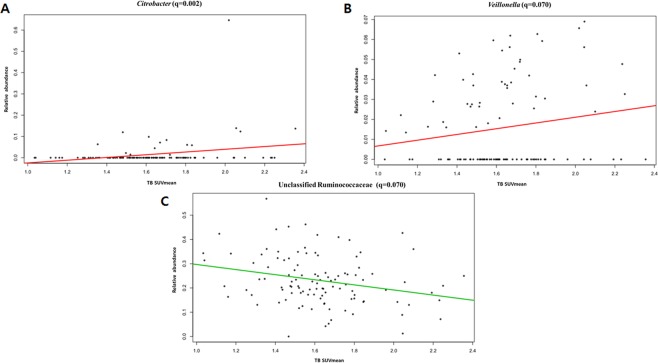
Correlations of the TB SUV_mean_ with *Citrobacter* (**A**), *Veillonella* (**B**), and the unclassified Ruminococcaceae (**C**).

In the analysis of the correlation between TB SUV_mean_ and gut microbial abundance, after adjustment of age and BMI, bacterial species of the genus *Citrobacter* (*CE* = 3.38; *p* < 0.001; *q* = 0.002; Fig. 4A) and the genus *Veillonella*, which belongs to the family *Veillonellaceae*, showed a positive correlation trend with TB SUV_mean_ (*CE* = 0.657; *p* < 0.001; *q* = 0.070; Fig. 4B). Additionally, the abundance of Ruminococcaceae was negatively correlated with the TB SUV_mean_ (*CE* = −0.729; *p* < 0.001; *q* = 0.070; Fig. 4C).

### Association between pro-inflammatory cytokine level and intestinal FDG uptake

The overall serum concentrations of TNF-α and IL-1 of all 114 patients were 0.336 ± 0.077 pg/mL (range = 0.268–0.929 pg/mL) and 0.483 ± 0.214 pg/mL (range = 0.321–2.714 pg/mL), respectively. The levels of TNF-α were significantly increased in the high IU group compared to the low IU group (0.33 ± 0.08 [low IU] vs. 0.34 ± 0.06 [high IU], *p* = 0.017). Furthermore, TNF-α levels were positively correlation with the TB SUV_max_ (*rho* = 0.220 and *p* = 0.018), as well as TB SUV_mean_ (*rho* = 0.250 and *p* = 0.007). However, serum IL-1 level showed no significant relationship with the visually graded IUs (0.47 ± 0.16 [lower IU] vs. 0.49 ± 0.29 [higher IU], *p* = 0.382), as well as with the quantitatively measured TB SUV (*rho* = −0.054 and *p* = 0.566 [TB SUV_max_]; *rho* = −0.032 and *p* = 0.737 [TB SUV_mean_]).

## Discussion

This study investigated the correlation between physiologic intestinal uptake (IU) and the composition of the gut microbiome in breast cancer patients. In this study, we found the differences in beta diversity to be marginally significant between lower and higher IU groups. The physiologic IU was positively correlated with the relative abundance of the genus *Citrobacter*, while negatively correlated with the unclassified Ruminococcaceae.

The potential role of gut microbiota in intestinal ^18^F-FDG uptake visualized on FDG PET was initially raised from the study by Franquet *et al*.^1^. They found that there was a significant reduction in intestinal ^18^F-FDG uptake with pretreatment with rifaximin, an antibiotic agent retained in the intestinal lumen. They concluded that this reduction in uptake might reflect a reduction in luminal bacterial burden or function in response to rifaximin therapy. However, a mechanism to explain the transfer of ^18^F-FDG from the intravascular compartment to the gut lumen has not yet been identified. Conversely, another recent study reported an increase of intestinal ^18^F-FDG uptake after oral administration of the broad-spectrum antibiotic vancomycin^3^. They demonstrated an increase in intestinal ^18^F-FDG uptake in all participants following vancomycin treatment that results in a four- to five-log reduction in gut bacterial load. They explained the phenomenon as a shift in colonocyte metabolism from lipolysis of single chain fatty acid (mainly butyrate), normally produced by the gut microbiota, to glycolysis.

In this study, we observed a negative correlation between the intestinal ^18^F-FDG uptake and the abundance of the bacterial  unclassified Ruminococcaceae. The low abundance of the Ruminococcaceae has been reported in several inflammatory disorders^19,20^. Ruminococcaceae contributes to butyrate production, which is the main microbial-derived gut mucosal immunity regulator and the best indicator of a healthy, mature anaerobic gut microbiota^6^. Butyrate-producing bacteria have recently gained attention because of their importance in the maintenance of a healthy colon, and loss of this group of bacteria is associated with the development of inflammatory bowel disease, Crohn’s disease, and ulcerative colitis^21^. Kang *et al*. also reported that an increase in intestinal ^18^F-FDG uptake was associated with low relative abundance of the unclassified Clostridiales, which including the family Ruminococcaceae, in healthy subjects. They suggested that the mucosal inflammation, impaired barrier function and gut permeability caused by microbial dysbiosis induced the uptake of intestinal ^18^F-FDG. Thus, an increase in intestinal ^18^F-FDG uptake may reflect a low abundance of butyrate-producing gut microbiota, mucosal disruption and be an early indicator for an increased risk of bowel disease.

We also found a trend of higher relative abundance of the *Citrobacter spp*. in high IU group, and a significant positive correlation with quantitatively measured TB SUV_max_ and SUV_mean_. The *Citrobacter spp*. is an aerobic gram-negative rod often found in the human intestine. The species rarely give rise to serious infections, but are opportunistic pathogens that can lead to bacteremia^22^. Of the 12 recognized species within the genus *Citrobacter*, *C. freundi* is known to cause abnormal inflammatory changes in the intestinal tract^22^. Moreover, *C. freundi* has been suspected to cause diarrhea and extra-intestinal peritonitis^23^. Several studies have reported the relationship of *Citrobacter* with irritable bowel syndrome.

The group *Veillonellaceae* showed a positive correlation trend with TB SUV_mean_. The relationship of the *Veillonellaceae* with a high-fat diet and obesity has been reported in previous studies^24,25^. We previously showed that there is a significant relationship between intestinal ^18^F-FDG uptake and obesity and lipid metabolism^4^. However, in this study, we did not find any relation between intestinal ^18^F-FDG uptake and obesity or lipid metabolism. The differences in the distribution of BMIs and lipid profiles between the two studies may be the cause of this discrepancy.

In addition to obesity, *Veillonellaceae* has previously been suggested to be a biomarker for inflammatory bowel disease^26^. A strong relationship between the increased abundance of *Veillonellaceae* bacteria and the occurrence of Crohn’s disease has been reported in a large cohort of pediatric patients^27^. Recently, the *Veillonellaceae* family was shown to promote a beneficial microenvironment for the progression of colorectal carcinoma^28^. Though we did exclude patients with inflammatory bowel disease from this analysis, there may have been subclinical inflammation of the intestine in some cases.

When the relationship between the pro-inflammatory cytokine TNF-α with intestinal ^18^F-FDG uptake was investigated, the level of TNF-α was significantly positively correlated with visually graded and quantitatively measured intestinal ^18^F-FDG uptake (both of TB SUV_max_ and TB SUV_mean_). Increased levels of this pro-inflammatory cytokine could be a biomarker for subclinical inflammation. An important role of TNF-α as a pivotal pro-inflammatory mediator, especially in inflammatory bowel disease, has been emphasized for decades^29^. Based on the significant relationship of intestinal ^18^F-FDG uptake with TNF-α, we believe that intestinal ^18^F-FDG uptake visualized on PET can be used as an imaging biomarker for subclinical mucosal inflammation.

Unlike TNF-α, IL-1 showed no significant relationship with intestinal ^18^F-FDG uptake. Regarding the role of the IL-1 family in intestinal inflammation, both pro-inflammatory and protective properties have been described. Bersudsky *et al*. demonstrated that, in a study of the development of colitis in mice, IL-1α played a pro-inflammatory role and that IL-1β had a protective effect on disease progression^30^. One of the limitations of this current study is that only the levels of IL-1β were measured. Further studies are therefore needed to determine the relationship between IL-1α and intestinal ^18^F-FDG uptake.

In this study, we found that the physiologic intestinal uptake of ^18^F-FDG was associated with changes in the abundance of several groups of intestinal bacteria in breast cancer patients. Though the specific intestinal bacteria were different from the study by Kang *et al*.^2^, which was performed in healthy subjects, the significant positive correlation between background IU with the genus *Citrobacter* and *Veillonella* imply that intestinal FDG uptake could potentially be used as a marker of mucosal inflammation. The negative relationship of butyrate-producing gut microbiota with intestinal ^18^F-FDG uptake seen in previous studies was replicated in our study. Moreover, we demonstrated that the is a significant correlation between the pro-inflammatory TNF-α cytokine and intestinal ^18^F-FDG uptake, which further supports the link between mucosal inflammation and physiologic intestinal uptake.

The major limitation of this study is that a direct comparison of gut microbiota between healthy subjects and cancer patients was not performed. Future work should include a comparison of the metagenomes of breast cancer patients with that of the healthy subjects in order to determine whether these findings should be limited to cancer patients or can be expanded to the general population. Secondly, we used fecal material for metagenome analysis. It has been reported that mucosal samples may be more indicative of significant relationships than fecal samples, indicating that the bacteria residing in the mucosal layer may play a more important role in disease etiology^27^. However, obtaining mucosal samples is more difficult and requires more invasive procedures than fecal samples, which can be a limiting factor in a clinical setting. Third, we did not check each patient’s bowel habit. Whether bowel habits affect intestinal FDG uptake is controversial. Yasuda *et al*. suggested that the presence of constipation affected intestinal FDG uptake^31^, while Kim *et al*. reported that there was no significant difference in intestinal FDG uptake score according to bowel habits^32^. They found that intense focal uptake pattern was observed in the descending colon of constipation group and such a focal FDG accumulation was caused by stool radioactivity. In this study, we measured the physiologic intestinal FDG uptake by placing VOI to avoid focal radioactivity of stool by simultaneous review of PET and CT slice. Thus, the effect of bowel habits on background IU will be very small.

## Supplementary information

Supplement Figure S1
