# Supplementary material for: Physiologic intestinal 18F-FDG uptake is associated with alteration of gut microbiota and proinflammatory cytokine levels in breast cancer
Source: Sci Rep. 2019 Dec 4;9:18273. doi: 10.1038/s41598-019-54680-3 (PMC6892830; doi:10.1038/s41598-019-54680-3)
Supplement: Supplementary file 1 — Supplement Figure S1 [file 41598_2019_54680_MOESM1_ESM.docx]

**Physiologic intestinal ^18^F-FDG uptake is associated with alteration of gut microbiota and proinflammatory cytokine levels in breast cancer**

Hai-Jeon Yoon^1‡^, Han-Na Kim^2,3‡^, Ji-In Bang^1^, Woosung Lim^4^, Byung In Moon^4^, Nam Sun Paik^4^, Bom Sahn Kim^1*^, Hyung-Lae Kim^5*^

^1^Department of Nuclear Medicine, Ewha Womans University School of Medicine, Seoul, Republic of Korea

^2^Medical Research Institute, Kangbuk Samsung Hospital, Sungkyunkwan University, School of Medicine, Seoul, Republic of Korea

^3^Department of Clinical Research Design and Evaluations, SAIHST, Sungkyunkwan University, Seoul, Republic of Korea

^4^Department of Surgery, School of Medicine, Mokdong Hospital, Ewha Womans University, Seoul, Republic of Korea

^5^Department of Biochemistry, Ewha Womans University, School of Medicine, Seoul, Republic of Korea

‡Hai-Jeon Yoon and Han-Na Kim evenly contributed to this work.

* Bom Sahn Kim and Hyung-Lae Kim evenly contributed to this work.


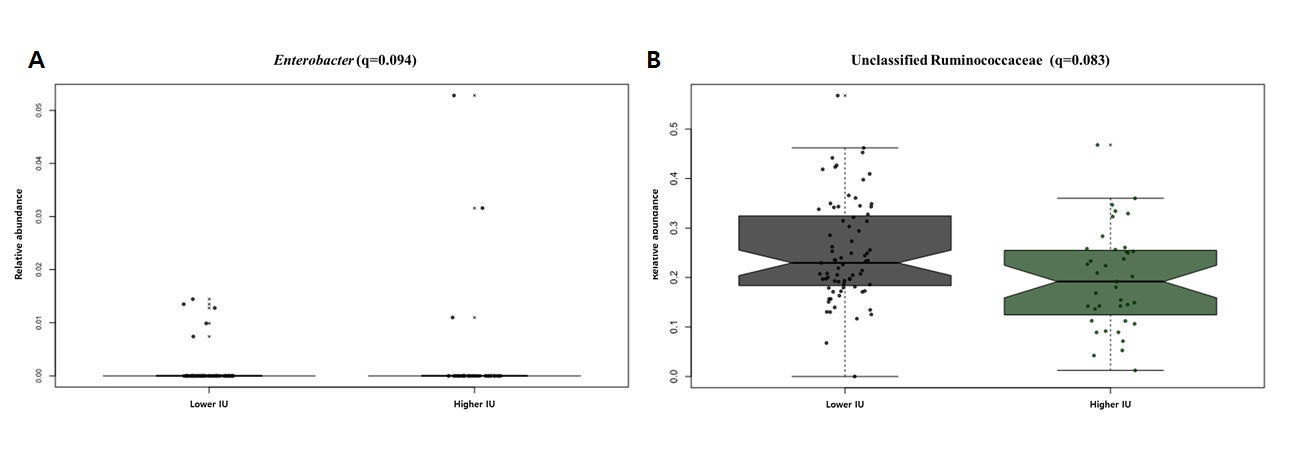


**Supplement Figure S1.** The relative abundance of *Enterobacter* (A) and the unclassified Ruminococcaceae (B) in each physiologic intestinal FDG uptake (IU) group.
